# Antenatal Diagnosis of Alobar Holoprosencephaly

**DOI:** 10.1155/2014/724671

**Published:** 2014-07-14

**Authors:** Rajesh Raman, Geetha Mukunda Jagadesh

**Affiliations:** Department of Radiodiagnosis, JSS Medical College, Mysore, Karnataka 570004, India

## Abstract

A twenty-year-old second gravida presented to the department of radiodiagnosis for routine obstetric ultrasound examination. Ultrasonography revealed a live fetus of 17 weeks with absent falx, fused thalami, monoventricle, proboscis, and cyclopia. Fetal MRI was performed and the findings were confirmed. Even though ultrasonography is diagnostic in the detection of fetal anomalies, MRI plays a vital role due to its multiplanar capability and excellent soft tissue resolution. The importance of presenting this classical case of alobar holoprosencephaly is to sensitize the clinicians and radiologists to the imaging manifestations of holoprosencephaly and to stress the importance of early diagnosis. If diagnosed in utero at an early stage of pregnancy, termination can be performed and maternal psychological trauma of bearing a deformed fetus can be avoided.

## 1. Introduction

Holoprosencephaly is the result of absent or incomplete cleavage of the prosencephalon [1]. The prosencephalon forms the cerebral hemispheres, the thalami, and the basal ganglia. Hence abnormalities in the development of the prosencephalon result in variable fusion anomalies of these structures. The alobar variety is the most severe form [2] of holoprosencephaly and incompatible with life. Hence early diagnosis by fetal ultrasonography allows for early termination of pregnancy and avoids maternal psychological trauma of giving birth to a deformed fetus.

## 2. Case Report

A twenty-year-old second gravida presented to the department of radiodiagnosis for routine obstetric ultrasonography. Her first child was a girl and normal. There was no history of consanguinity of marriage. Her general physical examination was within normal limits. On clinical examination, the gestational age corresponded to 16–18 weeks.

**Figure 1:**
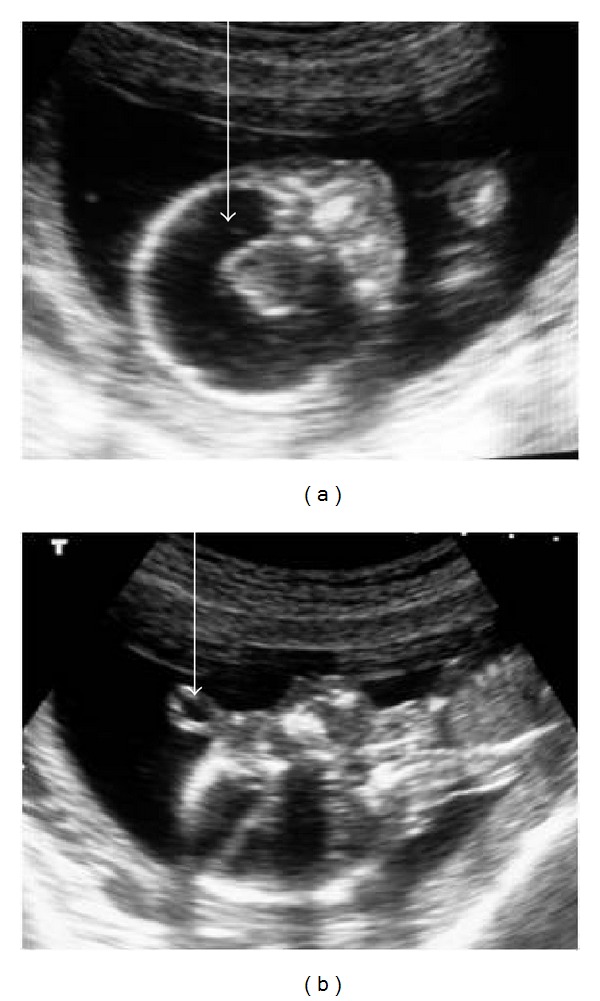
(a) Coronal ultrasound image showing the fused thalami in the centre and large monoventricle (thick white arrow). The amniotic fluid around the fetus is normal in quantity. (b) Sagittal ultrasound image showing the proboscis (thin white arrow) as a tubular cystic projection in the frontal region.

**Figure 2:**
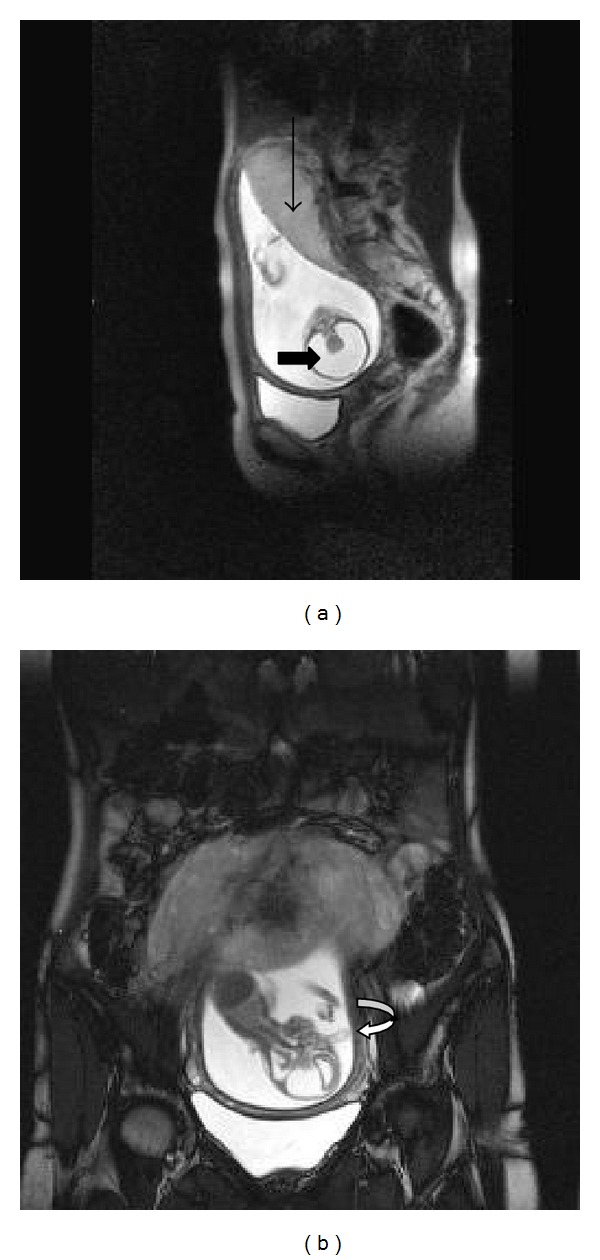
(a) T2 weighted image in the sagittal plane showing fused thalami and large monoventricle (short black arrow). Placenta is seen in the fundus (long black arrow). (b) T2 weighted image in the coronal plane showing the proboscis (curved black arrow). The brain stem and the spinal cord appear normal.

Ultrasonography revealed single live intrauterine fetus with an average gestational age corresponding to 17 weeks. The fetal skull bones were poorly ossified. The supratentorial brain was replaced by CSF with a thin rim of peripheral cerebral parenchyma and a large central monoventricle, with fused thalami in between (Figure 1(a)). The falx cerebri and septum pellucidum were not visualized. There was an external median tubular soft tissue projection (proboscis) in the face, at the level of the frontal bones without intracranial extension (Figure 1(b)). The facial structures were dysmorphic with fused orbits and single horizontal median eye in the forehead (cyclopia). The spine, thoracic cage, heart, and limbs were sonologically normal. The posterior fossa structures were normal. The umbilical cord and rest of the fetal organs were normal. Polyhydramnios was not present. Fetal MRI performed on 1.5 T MRI machine confirmed the sonographic findings (Figures 2(a) and 2(b)). There was no evidence of polyhydramnios or any other associated anomaly on MRI.

**Figure 3:**
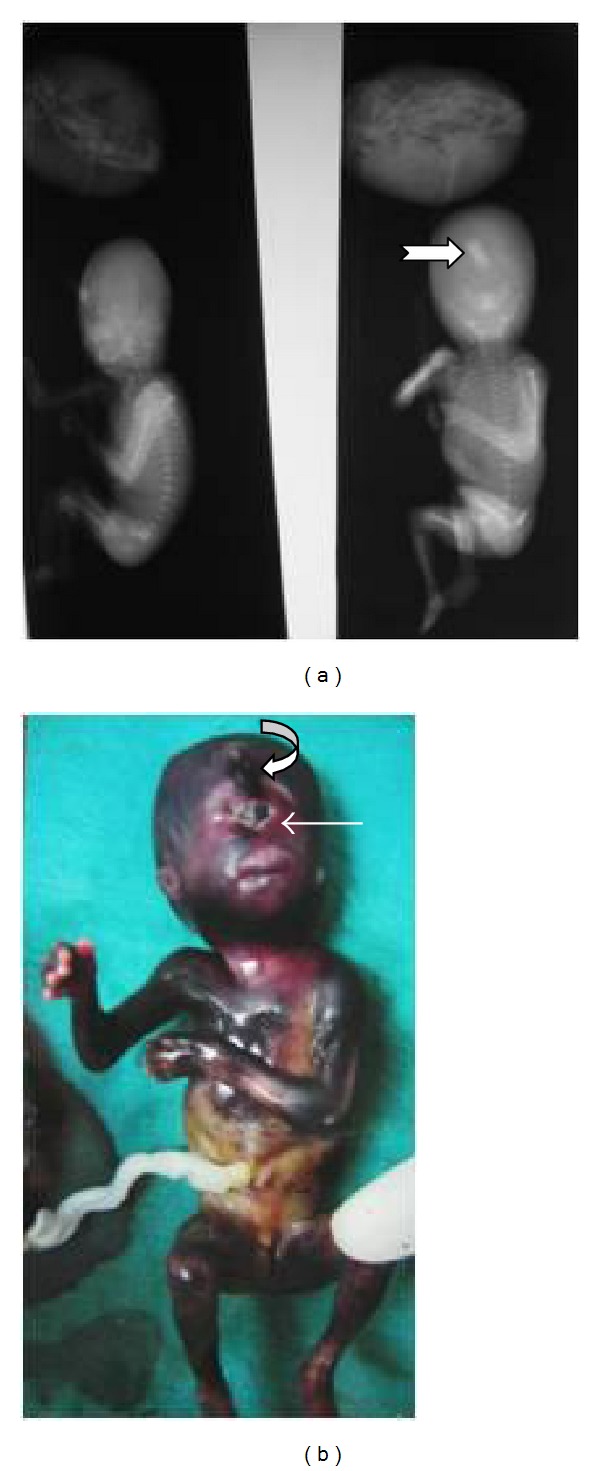
(a) Radiograph of the specimen with the placenta showing the proboscis (notched white arrow) as a dense structure in the frontal region and normal spine. (b) Photograph of the specimen showing facial dysmorphism, proboscis (curved white arrow), and cyclopia (straight white arrow).

The pregnancy was terminated and the gross specimen of the fetus (Figure 3(b)) showed all the findings observed on imaging. In addition, there was polydactyly involving all the four limbs. Plain radiography of the specimen (Figure 3(a)) was performed along with the placenta. It showed poor ossification of the skull bones, median proboscis, and normal spine.

Thus, this is a classical case of alobar holoprosencephaly with facial dysmorphism. The purpose of publishing this case is to sensitize the clinicians to the classical features of holoprosencephaly on various imaging modalities and to stress the importance of its detection before 20 weeks of gestation so as to allow for legal medical termination.

## 3. Discussion

Holoprosencephaly is a spectrum of cerebrofacial anomalies resulting from the complete or partial failure of the diverticulation and cleavage of the primitive forebrain [2, 3]. During the 4th gestational week, the neural tube forms the three primary brain vesicles, namely, prosencephalon, mesencephalon, and rhombencephalon. By the 5th week of intrauterine life, the prosencephalon further divides into the telencephalon and diencephalon. The telencephalon forms the two cerebral hemispheres whereas the diencephalon forms the thalami, the hypothalamus, and the basal ganglia.

The prechordal mesoderm takes part in the formation of the midline facial structures. The degree of facial dysmorphism is proportional to the severity of the intracranial abnormalities and should direct the sonologist to search for the CNS anomalies. This has led to the popular statement “face predicts the brain” by DeMeyer [4].

Our case showed two of the classical facial anomalies, namely, cyclopia (absence of the normal eye balls and a median horizontally placed eye in the fore head) and proboscis (median primitive noncanalized nose as a projection from the forehead). Cyclopia is the most severe abnormality amongst the median cerebrofacial anomalies [5] and mild hypotelorism with flat face is the least severe [6]. Sonography can detect up to 58% of the facial abnormalities [6].

Cebocephaly (monkey like head, deformed nose, and severe hypotelorism), ethmocephaly (cyclopia with deformed displaced nose), midline cleft lip, lateral cleft lip, and mild hypotelorism without cleft lip are the other facial abnormalities associated with holoprosencephaly [6]. The embryonic forebrain is responsible for induction of proper development of the orbits [7]. Thus, improper diverticulation and cleavage of the forebrain results in improper induction of the formation of orbits and leads to cyclopia [7]. Deftereou et al. described the morphological, radiographic, and immunohistochemical findings in a case of cyclopia [7]. However, immunohistochemistry was not performed in the present study.

There are three main forms of holoprosencephaly, namely, alobar, semilobar, and lobar varieties. The alobar holoprosencephaly is the most severe form and shows undifferentiated holosphere of the cerebral parenchyma with a central monoventricle and fused thalami [3]. The falx, interhemispheric fissure, corpus callosum, optic tracts, olfactory bulbs, and the septum pellucidum are absent [2]. Absence of septum pellucidum may be associated with septooptic dysplasia, holoprosencephaly, corpus callosal agenesis, schizencephaly, Chiari-II malformation, hydranencephaly, porencephaly, and cephaloceles. In a study of 2007 patients, Barkovich and Norman have described the above abnormalities along with absent septum pellucidum [8]. In our patient also, septum pellucidum was absent.

A dorsal cyst may be observed in the posterior cranial fossa in very severe forms of holoprosencephaly [2] and some of these cases may also be associated with Dandy Walker malformation [1], agyria, polymicrogyria, and heterotopias [5]. Extra cranial anomalies like limb anomalies, polydactyly, lung hypoplasia, cardiac anomalies, renal dysplasia, omphalocele, hydrops fetalis, esophageal atresia, bladder exstrophy, and gastrointestinal or abdominal anomalies [2, 6] may also be observed. Our case did not show any such association.

Optimal sonographic view for evaluating the fetal face is the coronal view with the orbits, maxilla, and anterior mandible in one plane [9]. Three-dimensional ultrasonography (3D US) acts as a supplement to 2D ultrasonography in the evaluation of fetal craniofacial abnormalities [10]. The facial anomalies may not be clearly visible if the fetus is in occipitoanterior position [9].

Alobar holoprosencephaly can be differentiated from hydrocephalus by the presence of midline echogenic falx, absent septum pellucidum, separated thalami, and distinct lateral ventricles in the latter [1]. Hydranencephaly may also demonstrate absence or deviated falx but the thalami are not fused in this condition [1]. In both hydranencephaly and Dandy Walker malformation, the falx cerebri, interhemispheric fissure, corpus callosum, and 3rd ventricle are present [3].

The semilobar holoprosencephaly is of intermediate severity with early midline differentiation and sagittal separation [9]. It shows a rudimentary falx, partial interhemispheric fissure, absent septum pellucidum, partial separation of thalami, and large H-shaped monoventricle. The basal ganglia show variable fusion. The facial anomalies are mild, namely, cleft lip, cleft palate, and hypotelorism.

The mildest variety of holoprosencephalies is the lobar holoprosencephaly characterized by near total cleavage of cerebral hemispheres, presence of falx, interhemispheric fissure, and absent septum pellucidum [11]. The frontal horns appear squared off or box like due to the absence of septum pellucidum. The thalami and the basal ganglia are separated. It may be associated with minimal facial dysmorphism like hypotelorism. There is a fourth variant of holoprosencephaly called the middle hemispheric variant. In this condition, the interhemispheric fissure is formed in the frontal and occipital regions and absent in the parietal region with fusion of the hemispheres [11].

The alobar form of holoprosencephaly is incompatible with life. Children with semilobar, lobar, and middle hemispheric variants have variable survival. Those who survive present with seizures as one of the commonest manifestations [12]. Verrotti et al. have discussed the abnormalities in the molecules of cytoskeleton, signalling molecules, and molecules modulating glycosylation in the control of neuronal migration [12]. However, genetic analysis and karyotyping were not performed in our study.

The role of fetal MRI is in the confirmation of the sonographic findings and detection of any other additional anomaly. Postnatal MRI with diffusion fiber tractography may detect rare association of brain stem and long tract abnormalities in holoprosencephaly [11]. The lobar and middle hemispheric variants are not associated with significant abnormalities of the white matter tracts whereas the alobar and semilobar forms are associated with abnormalities of the medial lemniscus and the corticospinal tracts [11]. However, in our case, no other additional anomaly was detected on fetal MRI. Postnatal 3D CT may also be used for detailed evaluation of the craniofacial abnormalities in holoprosencephaly [13].

The aetiology of holoprosencephaly is unknown. There are a few theories citing the causes of mechanical, environmental, and genetic factors and infections. Association of cyclopia is observed with cytomegalovirus infection [14] and maternal ingestion of salicylates [15] even in the absence of holoprosencephaly. Reports of association of maternal diabetes [16] with holoprosencephaly are also available.

Fetal karyotyping is advisable in all the cases of holoprosencephaly as most of them are associated with chromosomal anomalies. Even though karyotyping is not necessary for the diagnosis of holoprosencephaly, it will surely have a role in the identification of translocations and in the genetic counselling for future pregnancies. In a study of 33 fetuses with trisomy 13, Lehman et al. [17] have detected holoprosencephaly in 13 fetuses (39%). Fifty-five percent of the fetuses with holoprosencephaly showed chromosomal abnormalities in the study of Nyberg et al. [1]. However, karyotyping was not performed in our case. Advanced investigations like fetal karyotyping may not be available in all the places. Diagnosis of holoprosencephaly before 20 weeks of gestation by imaging is essential in order to avoid the psychological pain of bearing the deformed fetus till term and delivering a still born baby.
